# Cloning and Characterization of 12 TCP Genes in Medicinal Plant *Plantago asiatica* via De Novo Transcriptome Assembly

**DOI:** 10.3390/genes16091021

**Published:** 2025-08-28

**Authors:** Xingbin Lv, Ling Zhang, Yufang Hu, Tingting Jing, Qi Liang, Zhiyi Zhang, Mingkun Huang, Hua Yang

**Affiliations:** 1 Jiangxi Provincial Key Laboratory of Ex Situ Plant Conservation and Utilization, Lushan Botanical Garden, Chinese Academy of Sciences, 9 Zhiqing Road, Jiujiang 332900, China; 2 School of Pharmacy, Jiangxi University of Chinese Medicine, 1688 Meiling Avenue, Xinjian District, Nanchang 330004, China; 3 Nanchang University, 999 Xuefu Avenue, Honggutan New District, Nanchang 330047, China; 4 Guangdong Provincial Key Laboratory of Applied Botany & State Key Laboratory of Plant Diversity and Specialty Crops, South China Botanical Garden, Chinese Academy of Sciences, Guangzhou 510650, China

**Keywords:** *Plantago asiatica*, TCP, transcription factor, transcriptome, medicinal plant

## Abstract

**Background:** *Plantago asiatica* (*P. asiatica*) is an important Chinese traditional medicinal plant of the family Plantaginaceae and widely used in pharmaceutical industries. TCP transcription factors play an important role in plant development, but a limited number of studies on this have been reported in *P. asiatica.* **Methods:** Since genome assembly was not available, in this study, we used the de novo transcriptome assembly method to genome-wide-characterize the TCP gene family in *P. asiatica*. Up to 70.7 M high-quality paired-end reads were generated after sequencing and a total of 12 TCP genes were cloned by the predicted bioinformatic results, which were named *PaTCP1-12*. **Results:** Phylogenetic tree, motif analysis and subcellular localization results revealed that these *PaTCPs* were conserved compared to those from the model plant, *Arabidopsis*. Expression analysis suggested that most of the TCPs were highly expressed in both the leaf and root, while *PaTCP1*, *PaTCP6* and *PaTCP9* could also be detected in the seed. **Conclusions:** Since seed characteristics are one of the main agronomical traits in *P. asiatica*, the finding of *PaTCP1*, *PaTCP6* and *PaTCP9* expression patterns in the stem suggested an important role for further plant improvement.

## 1. Introduction

*P. asiatica*, belonging to Plantaginaceae [1], is not only a traditional medicinal plant but also serves as a nutritious vegetable or industrial material in Asia [2,3]. As for herbal medicine, bioactive compounds such as flavonoids, polysaccharides and alkaloids originate from seeds and leaves [1], which exhibit a wide range of pharmaceutical effects, such as antioxidant [4,5], anti-inflammatory [6], antibacterial [7], antiviral [8], and anticancer properties [9]. Besides these medical functions, *P. asiatica* is also used for industrial purposes. For example, it has been used as a deflocculant in paper and textile manufacturing, as an emulsifying agent, as a binder or lubricant in meat products, and as a replacement for fat in low-calorie foods. It has also been incorporated into breakfast cereals, ice creams, instant beverages, baked goods, and other dietary products [10,11]. Due to its economic and medicinal importance, molecular studies on *P. asiatica* remain scarce, mainly due to the lack of a reference genome. Consequently, little is known about the transcriptional regulatory networks controlling its growth, development, and metabolite biosynthesis [12,13]. Interactions between transcription factors (TFs) and their *cis*-regulatory elements (CREs), along with modulation by microRNAs (miRNAs), are key determinants of gene regulation [14,15,16]. TFs, for instance, are proteins that bind to specific DNA regions like promoters to activate or repress transcription, a regulatory mechanism that is essential for plant development and responses to abiotic stress [17]. RNA-Seq (transcriptome sequencing) has proven invaluable for revealing these functional genes and their interactions in diverse environments, and it holds great potential for plant breeding, the study of genetic variation, and understanding the synthesis and regulation of pharmacologically active substances [18].

Among various transcription factor families, the TEOSINTE BRANCHED 1/CYCLOIDEA/PROLIFERATING CELL FACTOR (TCP) family has attracted considerable attention. TCP transcription factors, a plant-specific family, are characterized by a conserved 59-amino-acid TCP domain, a noncanonical basic helix–loop–helix (bHLH) structure essential for DNA binding and protein–protein interactions [19,20]. Based on phylogenetic analysis and slight differences in TCP domains, TCPs are divided into two major classes (I and II), with Class II further divided into the CINCINNATA (CIN)-like and CYCLOIDEA/TEOSINTE BRANCHED 1 (CYC/TB1)-like clades [21,22]. In most plants, the TCP genes have expanded through evolution and differentiation processes, leading to a significant abundance of these genes in species like *Arabidopsis thaliana* (24) [23], *Glycine max* (55) [24], and *Brassica napus* (76) [25]. Concurrently, TCPs have evolved multiple sophisticated ways to precisely control their downstream factors, while their own expression is also tightly regulated. Recently, extensive studies have reported that TCPs are not only critical regulators in the development of roots, stems, leaves, flowers, fruits, and vascular systems, but also participate in important physiological processes such as secondary metabolism and responses to biotic and abiotic stresses [26].

Despite these important functions, TCP transcription factors have not yet been systematically identified or characterized in *P. asiatica*. This represents a critical knowledge gap because seeds and leaves—the plant’s primary medicinal organs—are also tissues where TCPs are expected to exert regulatory functions. Therefore, in this study, we performed a de novo transcriptome assembly of *P. asiatica* to identify TCP family members, clone their coding sequences, and characterize their conserved motifs, phylogenetic relationships, subcellular localization, and expression patterns across roots, leaves, and seeds. Our findings provide the first insight into the TCP gene family in *P. asiatica* and establish a foundation for understanding their potential regulatory roles in development and secondary metabolism.

## 2. Materials and Methods

### 2.1. Plant Material and Growth Conditions

*P. asiatica* used in this study was kept in our laboratory. The seeds were surface-sterilized with 70% ethanol for 30 sec and 10% NaClO for 10 min, then rinsed three times in sterile water and dried on sterilized filter paper. The treated seeds were sown on half-strength Murashige and Skoog (MS) plates containing 0.8% agar and 2% sucrose and then kept in the artificial climate chamber (22 °C, under a 16 h day/8 h night photoperiod) for germination. After one week, the young seedlings were transplanted to 15 cm diameter plastic pots with a 3:1 mixture of sterilized soil and vermiculite, at 28 °C, light/dark, 16/8 h with light intensity of 15,000 Lux in greenhouse conditions.

### 2.2. Transcriptome Library Preparation

Freshly harvested tissues (0.5 g) of *P. asiatica* (leaf, inflorescence, root, developing seed) were snap-frozen in liquid nitrogen. Total RNA was isolated using the RNeasy Plant Mini Kit (QIAGEN, Hilden, Germany), followed by poly(A)+ mRNA enrichment with Dynabeads™ Oligo(dT)25 (Invitrogen, CA, USA) from 5 μg of total RNA, according to the manufacturer’s protocol. The purified mRNA underwent first-strand cDNA synthesis using random primers. Library construction proceeded through second-strand synthesis, end repair, dA-tailing, and Y-adapter ligation (Vazyme Biotech, Nanjing, China), with final indexing via VAHTS Multiplex Oligos Set 4/5. Libraries were sequenced on an Illumina XTen platform (150 bp paired-end).

### 2.3. Transcriptome De Novo Assembly

Raw reads were processed with TrimGalore (http://github.com/FelixKrueger/TrimGalore, accessed on 2 February 2023, version 0.6.10) to remove adapters and low-quality bases (Phred score < 30, length < 35 bp). Clean reads were mapped against the Plant rRNA Database (https://www.plantrdnadatabase.com, accessed on 28 August 2025) to filter rRNA sequences. RNA-seq datasets passing quality thresholds were pooled for de novo transcriptome assembly using Trinity (https://github.com/trinityrnaseq/trinityrnaseq, accessed on 1 August 2024, version v2.15.2) with minimum k-mer coverage set to 4 (--min_kmer_cov 4). CD-Hit (https://github.com/weizhongli/cdhit, accessed on 1 May 2019, version v4.8.1) was used for filtering the redundant sequences. Open reading frames (ORFs) were identified using TransDecoder (https://github.com/TransDecoder, accessed on 16 July 2023, version v5.7.1).

### 2.4. Gene Annotation and Motif Analysis

Protein sequences of predicted ORFs were clustered via CD-HIT (https://github.com/weizhongli/cdhit) at 80% identity (-c 0.8) with word size 2 (-n 2), retaining cluster representatives. Non-redundant protein sequences were functionally characterized using eggNOG-mapper [27] for eggnog [28], KEGG [29] and GO [30] database annotations, with stringent thresholds (-value < 1 × 10^−5,^ bitscore > 50). Transcription factor classification was conducted using iTAK (http://itak.feilab.net/cgi-bin/itak/index.cgi) against the PlantTFDB database [31] under default parameters.

The distribution of GO terms across molecular function (MF), biological process (BP), and cellular component (CC) categories was statistically analyzed using TBtools (version 2.225) [32]. Non-redundant TCP transcription factors identified through TF annotation underwent de novo motif discovery using MEME Suite [33] with default parameters (width: 6-50 residues). Conserved motif patterns were mapped to TCP protein domains and visualized using TBtools’ advanced rendering module.

### 2.5. Phylogenetic Tree Analysis

A phylogenetic tree was reconstructed using MEGA11 (version 11.0.13) [34] with TCP protein sequences from *Arabidopsis thaliana* and *P. asiatica*. Multiple sequence alignment was performed using the MUSCLE algorithm, followed by Bayesian Information Criterion (BIC)-based model selection. The neighbor-joining tree was constructed with 1000 bootstrap replicates under the Jones–Taylor–Thornton (JTT) model [35].

### 2.6. PaTCPs Amplification and Quantitative PCR

Total RNA was extracted as described in Section 2.2. First-strand cDNA was synthesized from total RNA (extracted as described in Section 2.2) using M-MLV Reverse Transcriptase (Takara Bio, Shiga, Japan). Full-length *TfTCP* transcripts were amplified with specific primers (Table S1) KOD high-fidelity DNA polymerase (TOYOBO, Tokyo, Japan) and ligated into pMD19-T vectors (Takara Bio, Tokyo, Japan) for Sanger sequencing validation (Sangon, Shanghai, China).

Quantitative PCR (qPCR) was performed in triplicate with SYBR Premix Ex Taq™ (Takara) under the following conditions: 95 °C/30 s, 40 cycles of 95 °C/5 s, 60 °C/30 s. Relative expression levels were calculated using the 2^−ΔΔCq^ method [36], normalized to the reference gene *PaACTIN* (primers in Table S1).

### 2.7. PaTCPs Subcellular Localization

Full-length CDSs of *PaTCPs* (stop codons excluded) were directionally cloned into the 35S:GFP plant binary vector [37] using Gibson Assembly Master Mix (Voyzan, San Diego, CA, USA) to generate C-terminal GFP fusions, with specific primers (Table S1). Constructs were introduced into Agrobacterium tumefaciens strain GV3101:pSoup, and transiently expressed in tobacco epidermal cells via syringe infiltration [38]. Each assay was performed with three independent biological replicates. After 72 h incubation in growth chambers (23 °C, 16/8 h light/dark cycle), subcellular localization of fusion proteins was analyzed using confocal microscopy (LSM 710 NLO, ZEISS, Germany) with 488 nm excitation wavelength.

## 3. Results

### 3.1. De Novo Transcriptome Assembly of P. asiatica

Transcriptomic profiling of *P. asiatica* leaf, root and seed tissues generated 26.5 million (M), 23.1 M and 21.1 M raw paired-end reads via Illumina sequencing, respectively (Table S1). After rigorous Q30 filtering (base accuracy > 99.9%) and rRNA depletion through alignment against the Plant rDNA Database, 3.5 M (seed), 3.2 M (leaf) and 3.2 M (root) high-quality reads were retained for de novo assembly using Trinity (version v2.15.2) [39], which resulted in up to two hundred and nine thousand primary sequences, which were further subjected to the CD-Hit (version v4.8.1) software to remove the redundant sequences. The final filtered assembled sequences contained 59,728 unigenes (N50 = 1056 bp; mean length = 797.20 bp) (Table 1) and these unigenes were utilized for subsequent analysis.

### 3.2. Unigenes Annotation

**Figure 1:**
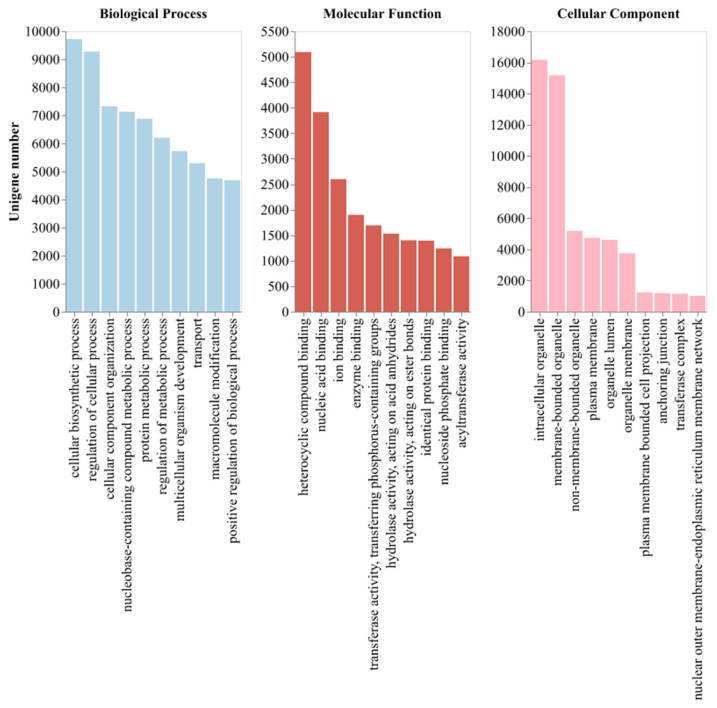
Gene Ontology (GO) classification of *P. asiatica* unigenes. The top 10 most abundant GO terms across molecular function, cellular component, and biological process domains are displayed.

Open reading frames (ORFs) of the 59,728 unigenes were predicted using TransDecoder (version v5.7.1). Among these, 50,684 sequences (84.86%) were functionally annotated by comparison with multiple databases, including UniProt, NR, GO, Pfam, KEGG, and COG; a summary of unigene annotation statistics is provided in Table 2. Specifically, 50,130 unigenes (83.93%) matched entries in the NR database (Table S3), and 36,934 (61.84%) showed homology in UniProt (Table S4). Functional categorization demonstrated 20,990 (35.14%) unigenes assigned to GO terms across molecular function, cellular component, and biological process domains (Figure 1, Table S5). Comparative domain analysis showed 37,606 (62.96%) Pfam matches (Table S5), while metabolic pathway annotations included 22,673 (37.96%) KEGG and 38,632 (64.68%) COG classifications (Table S5).

**Figure 2:**
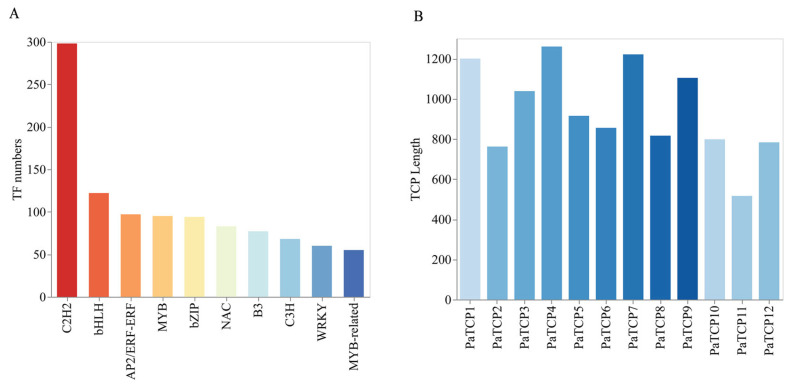
Prediction of transcription factor (TF) in *P. asiatica*. (**A**) Top 10 abundant TF families identified in *P. asiatica*, including AP2/ERF-ERF, C2H2, bHLH, WRKY, MYB, MYB-related, NAC, bZIP, GRAS, and C3H. (**B**) Coding sequence (CDS) length distribution of 12 validated *PaTCPs* genes in *P. asiatica*.

To identify transcription factors (TFs) in *P. asiatica*, unigene ORF sequences were analyzed with iTAK (version 1.6). We identified 1798 TFs classified into 66 families (Table S6), a distribution pattern consistent with angiosperm TF diversity reported in the PlantTFDB database. The ten largest families (C2H2, bHLH, AP2/ERF, MYB, bZIP, NAC, B3, C3H, WRKY, MYB-related) represented over 58% of all identified TFs, each comprising 55 to 298 unigenes (Figure 2A).

### 3.3. Clone and Sequencing Validation of the 12 TCP TF in P. asiatica

As mentioned above, TCP TFs might be involved in regulating plant development and considered as the genetic targets for plant improvement in genetic engineering [22,40]. We focused on the TCP TF in subsequent analysis. Based on the results of transcriptome assembly, a total of 19 TCPs were predicted. To validate these, we designed specific primers (Table S1) to amplify these TCP genes. Finally, 12 TCP TFs (named as *PaTCP1*–*PaTCP12*) were successfully isolated and validated by Sanger sequencing, with CDS lengths ranging from 516 bp (*PaTCP11*) to 1260 bp (*PaTCP4*) (Figure 2B, Table S7). Other TCPs were not able to amplify might because of incorrect assembly, which also suggested the genome of *P. asiatica* has high complexity.

### 3.4. Phylogenetic Tree and Motif Analysis of PaTCPs

**Figure 3:**
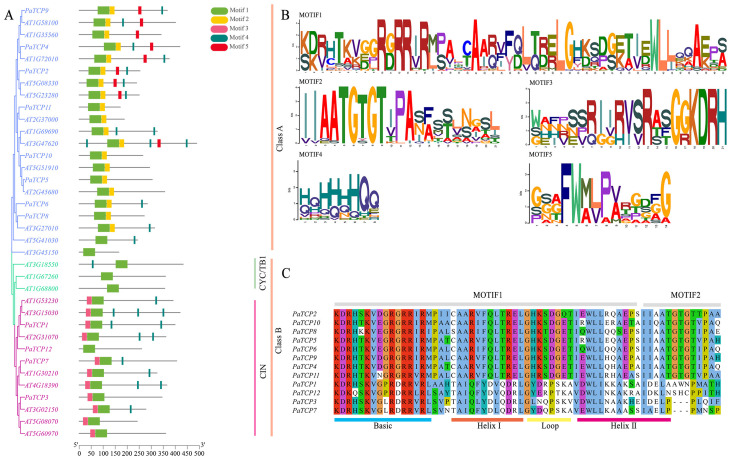
Phylogenetic tree and conserved motif analysis of *PaTCPs*. (**A**) Neighbor-joining phylogenetic tree constructed with 12 *P. asiatica PaTCPs* and 24 *A. thaliana AtTCPs*. (**B**) Conserved motifs (Motif1-5) identification in 12 PaTCP protein sequences. (**C**) The alignment of Motif1 and Motif2 used 12 PaTCP protein sequences.

Through phylogenetic tree construction, these 12 *PaTCPs* were phylogenetically divided into two major classes (Class I and Class II), through neighbor-joining analysis based on amino acid sequence alignment with 24 known *A. thaliana* TCPs (Figure 3A, left panel). As in previous reports [41], Class II was further categorized into two subclades: CINCINNATA (CIN) and CYC/TB1. Eight *PaTCPs* (*PaTCP2*, *4*, *5*, *6*, *8*, *9*, *10*, and *11*) were clustered into Class I, while the remaining four *PaTCPs* were exclusively grouped within the CIN subclade of Class II. In addition, we did not find any TCPs that were close to the CYC/TB1 subclade, which was reported to regulate floral symmetry [42,43]. This may be due to the incompleteness of transcriptome information and suggesting the importance of the full genome assembly.

To investigate conserved domain features of *PaTCPs*, we employed MEME Suite for motif analysis. Five conserved motifs (named Motif 1–5) were detected among *PaTCPs* (Figure 3A). Motif 1 contained signature residues (RD/GRRI/VR and WLLxxAxx) of the noncanonical bHLH TCP domain, which were conserved across both *PaTCPs* and *AtTCPs* in this study (Figure 3B,C). Motif 2 (Figure 3B,C) was also integral to the bHLH domain, exhibiting class-specific distributions. Motifs 2 and 5 were predominantly found in Class I TCPs, while Motif 3 was exclusive to the Class II CIN clade. Motif 4 is enriched in glutamine (Q) and histidine (H), which may be involved in protein dimerization [44].

These data suggested the conserved role of *PaTCPs*, facilitating further investigation of the function in plant development in *P. asiatica*.

### 3.5. Subcellular Localization Analysis of the PaTCPs

**Figure 4:**
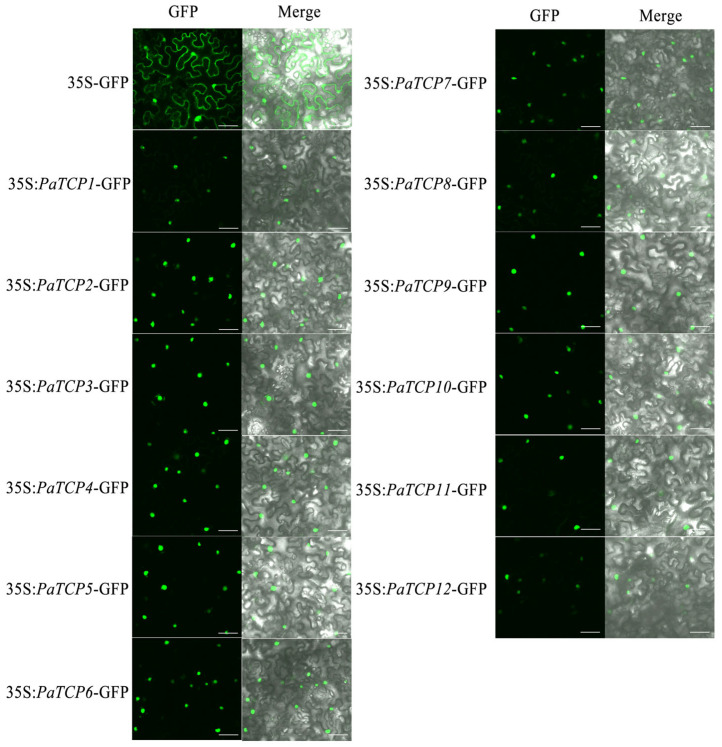
Subcellular localization of *PaTCPs* in *P. asiatica*. Confocal microscopy images of tobacco epidermal cells expressing 35S:GFP (empty vector control) and 35S:*PaTCPs*-GFP fusion proteins. Merged: GFP fluorescence (green channel) is overlaid on bright-field images. Scale bars = 50 μm.

TCP TFs are important regulators that can bind to specific motifs in the nucleus to regulate plant developmental processes, which suggested the subcellular localization of the TCPs should be in the nucleus. In *P. asiatica*, a total of 12 PaTCPs have been cloned as mentioned above. In order to confirm the nucleus localization of PaTCPs, we fused the PaTCPs upstream of the GFP protein and transformed them into tobacco leaf. As expected, all these 12 PaTCPs, including Class I and Class II, are exclusively localized in the nuclei (Figure 4). These data support the conserved function of PaTCPs, as in previous reports [45,46].

### 3.6. Tissue-Specific Expression of PaTCPs

**Figure 5:**
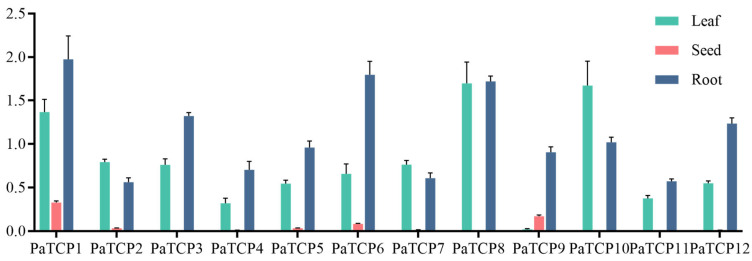
Tissue-specific expression patterns of *PaTCPs* in *P. asiatica*. Quantitative analysis of 12 *PaTCPs* across three tissues (leaves, roots, stems) using reverse-transcription quantitative PCR (RT-qPCR). Expression values were normalized against the constitutive control *PaACTIN*.

To delineate the tissue-specific expression patterns of PaTCPs, we conducted quantitative PCR (qPCR) analysis across three tissues (leaves, roots and seeds) in *P. asiatica* (Figure 5). Ubiquitous expression patterns were observed, with all of PaTCPs showing constitutive transcription in roots. Eleven out of these twelve PaTCPs (except for PaTCP9) were expressed highly in leaves, indicating their regulatory roles in leaf morphogenesis. Seed-enriched expression was uniquely detected in PaTCP1, PaTCP6 and PaTCP9, suggesting these three genes are involved in seed development.

## 4. Discussion

*P. asiatica* is an important Chinese traditional medicinal plant, but studies on its molecular biology remain limited, restricting further genetic research and potential improvement. In this study, we performed de novo transcriptome assembly combined with experimental validation to clone 12 PaTCP genes, and systematically investigated their sequence characteristics, phylogenetic relationships, subcellular localization, and tissue-specific expression patterns.

The results of phylogenetic and motif analysis revealed that PaTCPs can be divided into two major classes, consistent with TCP classification in other plant species such as *Arabidopsis* and *Chrysanthemum*. However, no members of the CYC/TB1 clade were detected, likely due to transcriptome incompleteness. This observation suggests that a genome assembly will be essential for a complete TCP family survey in *P. asiatica*.

Expression profiling demonstrated that these *PaTCPs* were involved in developmental progress. In particular, *PaTCP1*, *PaTCP6* and *PaTCP9* showed high expression levels in seeds, suggesting potential regulatory roles in seed development—an essential agronomic and medicinal trait of *P. asiatica*. Similar seed-related functions of TCP genes have been reported in other plants: for instance, *CnTCP9* in *Chrysanthemum nankingense* regulates seedling growth and size [47]. These findings imply that *PaTCP1*, *PaTCP6*, and *PaTCP9* may also influence both developmental and metabolic pathways in *P. asiatica*.

The de novo transcriptome assembly strategy has proven effective for uncovering key genes in other non-model plants such as *Torenia fournieri* and *Panax ginseng*. For example, in *Torenia fournieri*, TCP transcription factors are implicated in regulating leaf and floral morphology, while TCP genes in *Panax ginseng* have been associated with secondary metabolite biosynthesis [48]

Comparative analyses across species further highlight potential conserved and divergent functions of TCPs. While many PaTCPs share conserved motifs with AtTCPs, the unique expression signatures in *P. asiatica* seeds suggest possible lineage-specific adaptations. Future functional studies, including gene overexpression and silence experiments, will be required to validate their regulatory roles.

## 5. Conclusions

This study provides the first genomic resource for TCP transcription factors in *P. asiatica*. By identifying candidate TCP genes involved in seed and leaf development, our work lays a foundation for future research on genetic improvement and metabolic regulation in this medicinally and economically important species.

## Figures and Tables

**Table 1:** Statistical summary of the *P. asiatica* de novo transcriptome assembly.

Item	Value
Total number of filtered reads	70,762,701
Total length of filtered reads (bp)	47,612,229
Total numbers of unigenes	59,728
GC percentage (%)	45.75
N50 value of unigenes (bp)	1056
N90 value of unigenes (bp)	369
Mean length of unigenes (bp)	797.20
Median length of unigenes (bp)	537.00

**Table 2:** Statistical summary of unigene annotation in various databases.

Database	Number of Annotated Unigenes	Percentage
NR	50,130	83.93%
COG	38,632	64.68%
Pfam	37,606	62.96%
Uniprot	36,934	61.84%
KEGG	22,673	37.96%
GO	20,990	35.14%
TF	1798	3.01%
Total	50,684	84.86%

## Data Availability

The RNA-seq data of *P. asiatica* in this study were submitted to the National Genomics Data Center (https://ngdc.cncb.ac.cn, accessed on 28 August 2025) under the accession number PRJCA043284.

## Supplementary Materials

The following supporting information can be downloaded at: https://www.mdpi.com/article/10.3390/genes16091021/s1, Table S1: Summary of RNA-seq data and primer sequences used in this study; Table S2: Gene assembly; Table S3: NR annotation of unigenes; Table S4: Uniport annotation of unigenes; Table S5: Eggnog annotation; Table S6: TF annotation of unigenes; Table S7: Full length of TfTCP CDSs.
